# NLRP3: a new therapeutic target in alcoholic liver disease

**DOI:** 10.3389/fimmu.2023.1215333

**Published:** 2023-07-13

**Authors:** Subhashini Brahadeeswaran, Tiasha Dasgupta, Venkatraman Manickam, Viswanathan Saraswathi, Ramasamy Tamizhselvi

**Affiliations:** ^1^ Department of Biosciences, School of Biosciences and Technology, Vellore Institute of Technology, Vellore, Tamil Nadu, India; ^2^ Department of Internal Medicine, Division of Diabetes, Endocrinology, and Metabolism, Veterans Affairs Medical Center, University of Nebraska Medical Center, Omaha, NE, United States

**Keywords:** alcohol, NLRP3 inflammasome, epigenetics, alcohol liver disease, pyroptosis, interleukin 1β, interleukin 18, pro-caspase-1

## Abstract

The liver is in charge of a wide range of critical physiological processes and it plays an important role in activating the innate immune system which elicits the inflammatory events. Chronic ethanol exposure disrupts hepatic inflammatory mechanism and leads to the release of proinflammatory mediators such as chemokines, cytokines and activation of inflammasomes. The mechanism of liver fibrosis/cirrhosis involve activation of NLRP3 inflammasome, leading to the destruction of hepatocytes and subsequent metabolic dysregulation in humans. In addition, increasing evidence suggests that alcohol intake significantly modifies liver epigenetics, promoting the development of alcoholic liver disease (ALD). Epigenetic changes including histone modification, microRNA-induced genetic modulation, and DNA methylation are crucial in alcohol-evoked cell signaling that affects gene expression in the hepatic system. Though we are at the beginning stage without having the entire print of epigenetic signature, it is time to focus more on NLRP3 inflammasome and epigenetic modifications. Here we review the novel aspect of ALD pathology linking to inflammation and highlighting the role of epigenetic modification associated with NLRP3 inflammasome and how it could be a therapeutic target in ALD.

## 1 Introduction

Alcohol is a significant contributor to the global burden of preventable morbidity and mortality as well as a major risk factor for chronic diseases. An estimated 2.5 million people die from the harmful use of alcohol each year, a large portion of which is caused by alcoholic liver disease (ALD). ALD is demarcated as liver injury, inflammatory response, fibrosis, cirrhosis. In patients with other types of liver illness such as hepatitis C virus (HCV) infection, alcohol acts as a common co-factor and induces hepatic fibrosis (1, 2). About 25% of liver cirrhosis cases are known to have excessive alcohol exposure as their primary cause. Although almost everyone who is exposed to alcohol over time develops fatty liver (hepatic steatosis) which is the liver’s first reaction to alcohol usage, only people who drinks heavily for a prolonged period of time are at risk for developing severe liver diseases (3, 4).

Chronic ethanol exposure frequently accompanies liver damage due to altered alcohol dehydrogenase (ADH) and aldehyde dehydrogenase (ALDH) activities, the most important enzymes involved in the oxidation of ethanol, as well as increased cytochrome P450 family two subfamily E member 1 (CYP2E1) mediated ethanol metabolism (5). Ethanol is converted to acetyl-CoA in three steps, beginning with the oxidation of ethanol to acetaldehyde by alcohol dehydrogenase in the liver (6). The rate-limiting stage in alcohol metabolism is the conversion of acetaldehyde to acetic acid by aldehyde dehydrogenase 2. Eventually, acetic acid is transformed to acetyl-CoA and enters the citric cycle, where it can be released as H_2_O and CO_2_. During this process, alcohol and its metabolites affect the hepatic niche by increasing the release of reactive oxygen species (ROS), which acts as a signaling molecule to activate NF-κB, mitogen activated protein kinases (MAPK), and suppressor of cytokine signaling 3 (SOCS3) and, attenuate signal transducer and activator of transcription (STAT1 and 3). These signaling events promote the expression of proinflammatory cytokines such as IL-1β and TNF- α, thereby contributing to alcoholic hepatitis. Furthermore, ROS causes oxidative stress, mitochondrial damage, and activation of certain inflammasomes that are critically involved in hepatocellular damage associated with ALD ranging from steatosis, alcoholic steatohepatitis, fibrosis/cirrhosis to hepatocellular carcinoma (5, 7, 8).

Recent research has shown the crucial role of pyroptosis in ALD mediated by inflammasomes. The inflammasome is a multiprotein oligomer composed of caspase-1, an apoptosis-associated speck-like protein containing a caspase recruitment domain, and a pyrin domain from the NOD-like receptor family that mediates the response to cellular danger signals by stimulating and recruiting inflammatory cells (9). Inflammasome activates procaspase-1 which cleaves pro-IL-1β to its active form IL-1β (10). Till today, multiple types of inflammasomes have been identified such as NLRP1 (NLR Family Pyrin domain containing protein 1), NLRP2, NLRP3, NLRC4 (NLR family CARD domain-containing protein 4), NLRP6, NLRP7, NLRP12, IFI16 (Interferon Gamma Inducible Protein 16) AIM2 (absent in melanoma 2), and Pyrin (11). Among which, NLRP3 inflammasome has been well-defined in several inflammatory diseases. One of the major functions of inflammasome is to establish pyroptosis (unique inflammatory form of lytic programmed cell death) depending on effector enzyme, caspase -1/4/5 that facilitates the secretion of pro-inflammatory cytokines such as IL-1β and IL-18 (12). Increasing amount of evidence suggests that NLRP3 inflammasome activation is a key driver of variety of acute and chronic liver disorders. In hepatocytes, activation of NLRP3 causes pyroptotic cell death which contributes to liver injury (13). Alcohol is known to release danger signals like uric acid or extracellular adenosine triphosphate, all of which are NLRP3 inflammasome activators (14). Despite significant advancements in this area, the hunt for novel approaches is hampered by the lack of understanding of the NLRP3 inflammasome regulation processes. A new approach to comprehend the underlying mechanisms that control NLRP3 inflammasome activation and identifying new therapeutic targets has emerged from the research of the epigenetic mechanisms involved in regulating the NLRP3 inflammasome components. Immune cell growth, activation, and differentiation have all been linked to epigenetic control mechanisms, which are heritable alterations brought on by internal or external environmental stimuli that modulate gene expression without changing DNA sequence (15–18). Depending on the physiological or pathological situations in which they are found, these epigenetic alterations give cells the capacity to adapt and react to various stimuli (19). On the other hand ethanol and its metabolites (acetaldehyde, acetate, acetyl-CoA, and reactive oxygen species) may influence epigenetics by altering epigenetic enzyme activity, substrate availability for histone acetylation, DNA and histone methylation, and miRNA production (20). Thus, targeting epigenetics in ALD with existing Epidrugs may serve as a potential therapeutic strategy. Here, we review the current knowledge of NLRP3 inflammasome in ALD development, focusing on the inflammasomes-related epigenetic mechanisms, and summarizing the contribution of targeting NLRP3 epigenetic modification to therapy in ALD.

## 2 Role of NLRP3 inflammasomes in normal physiological condition

**Figure 1:**
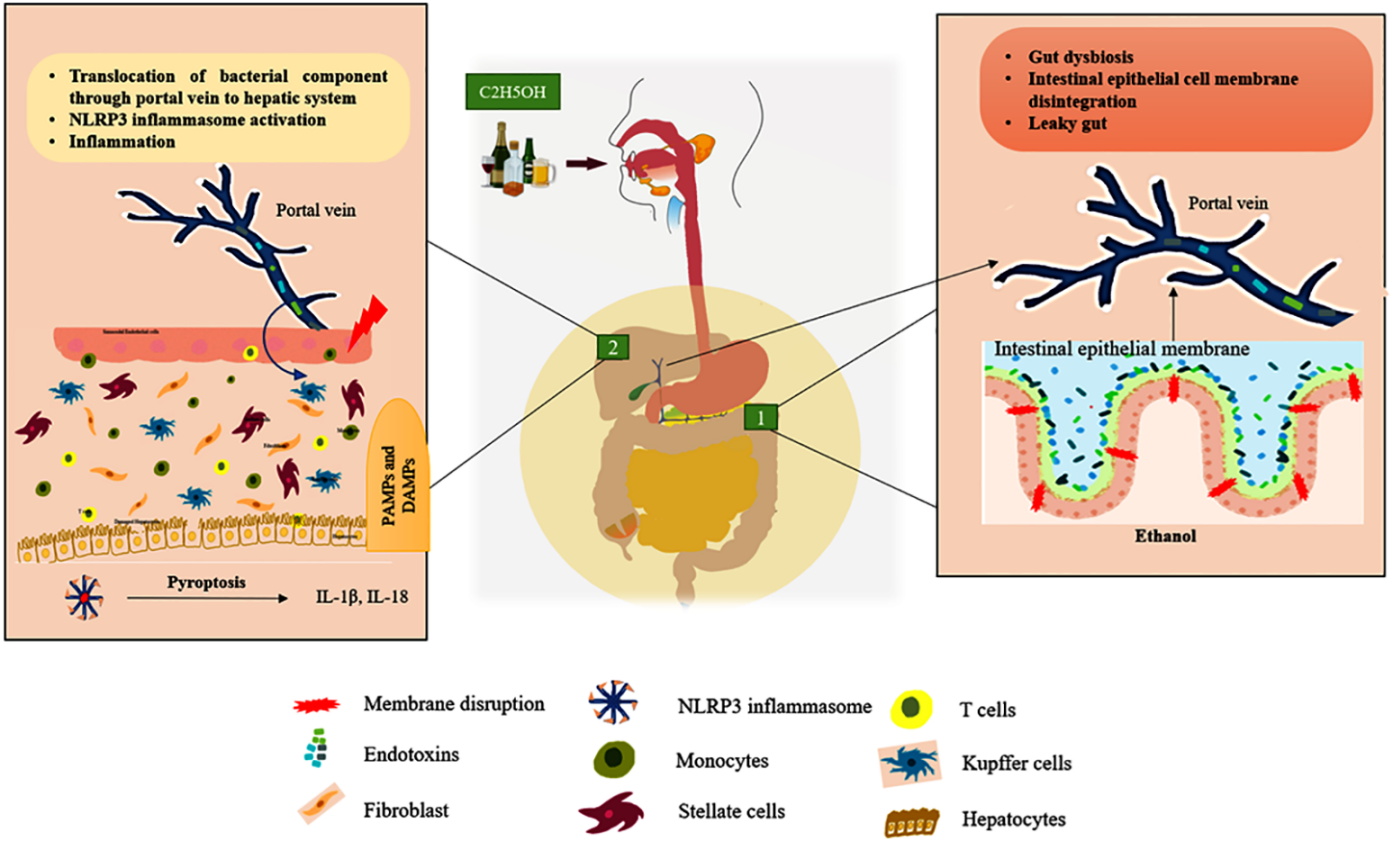
ALD’s gut-liver axis. Green box 1: Represent the chronic ethanol consumption develop the intestinal dysbiosis it releases endotoxins that breach the intestinal epithelial membrane and enter the portal vein, which connects the gut to the liver. Green box 2: Endotoxins (PAMPs) and other cellular damage mediated DAMPs enter the liver through the portal vein, activating cytosolic NLRP3 in tissue-resident cells to cause the pyroptosis process by secreting pro-inflammatory cytokines including IL-1β and IL-18. (Hepatocytes, Kupffer cells, and stellate cells are the resident cells of the liver tissue). In **Figure 3**, the activation of intracellular signaling is clearly shown.

Liver is responsible for the multi-tasking of metabolic reactions, detoxification, excretion, storage of vitamins and minerals, and immune response. Both the parenchymal (hepatocytes, stellate) and non-parenchymal (leukocytes, lymphocytes, and Kupffer cells) cells are involved in liver homeostasis (21). Within the context of any liver disease, both the parenchymal and non-parenchymal cells express the component of the inflammasome that modulates the *milieu* to undergo cellular injury. Through the portal vein connection, the gastrointestinal microbes invade the hepatic system and are recognized by the resident cells, leading to the activation of the innate immune response which is depicted in **Figure 1** (22). Intriguingly, the accumulation of these pathogen-associated molecular patterns (PAMPs) and damage-associated molecular patterns (DAMPs) enhances the activation of the inflammasome in the hepatic cells, facilitating the exacerbation of chronic liver diseases such as hepatic steatosis, fibrosis, and cirrhosis (23).

In normal physiological conditions, the components of an NLRP3inflammasome such as a sensor or pattern recognition receptor (nucleotide-binding oligomerization domain, leucine-rich repeat-containing proteins 3), an adaptor ASC (Apoptosis-associated speck-like protein containing a caspase recruitment domain) (24) and a zymogen pro-caspase-1 and 4/5 involved in both canonical (requiring both priming and activation/secondary signals) and non-canonical inflammasome cascades (activation signal binds directly to caspase) (25), respectively, are passive. The release of PAMPs and DAMPs via cell injury triggers the intracellular signaling cascade which, in turn, accelerates the complex formation of the inflammasome in tissue-resident cells.

**Figure 2:**
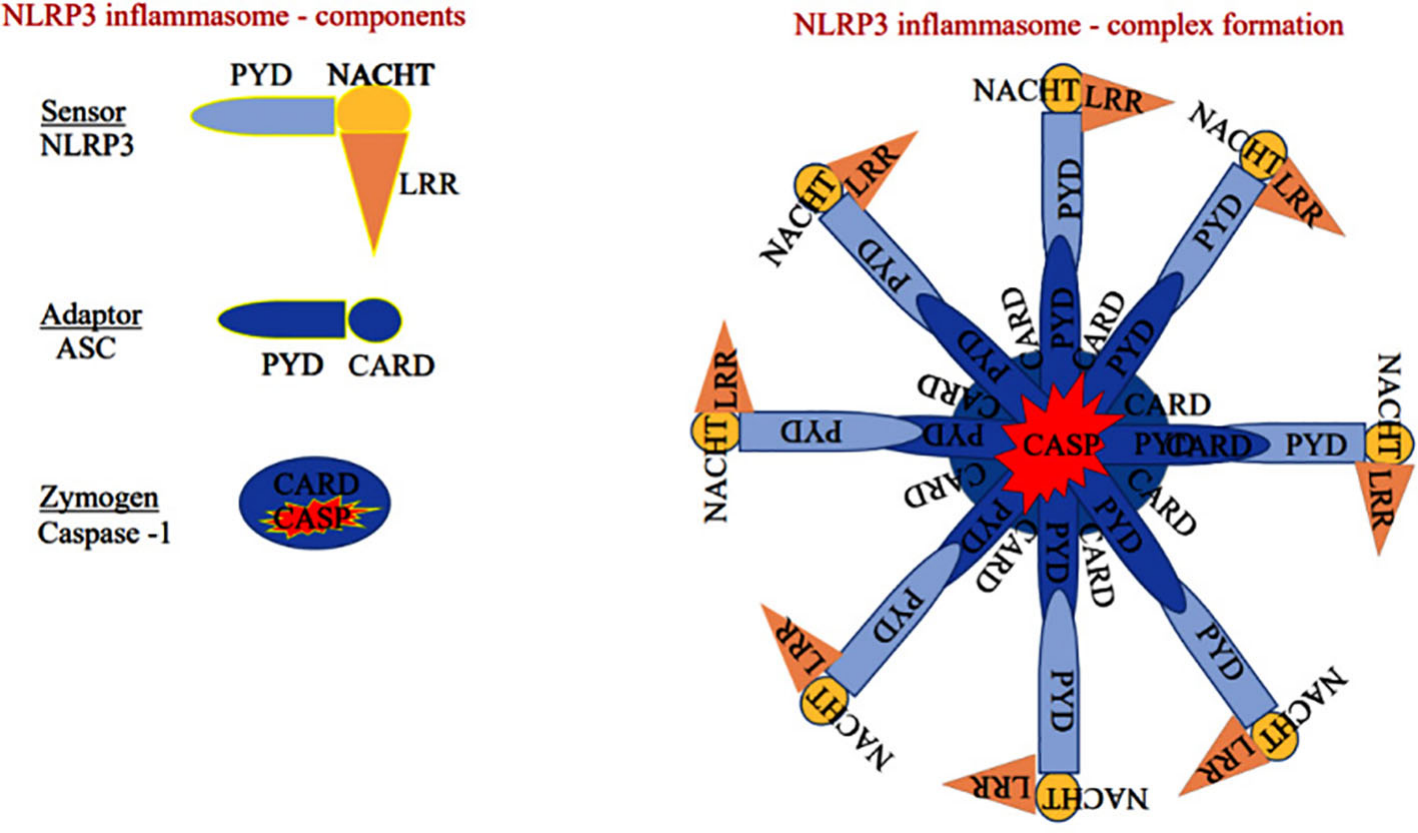
Sensor: Component of the NLRP3 inflammasome comprises of pyrin domain (PYD), nucleotide-binding oligomerization domain (NACHT), and leucine-rich repeat-containing proteins 3 (LRR), Adaptor: Apoptosis-associated speck-like protein containing a caspase recruitment domain (ASC) has pyrin and caspase activation and recruitment domain (CARD). Zymogen: Consists of CARD and caspase domain (either caspase-1 or 4/5).

NLRP3 monomer has been classically identified as a member of innate immunity, residing as an intracellular receptor comprised of three domains such as carboxy-terminal-leucine-rich repeats at the center, the Walker A and B motif containing the nucleotide-binding and oligomerization (NACHT) domain, and amino-terminal pyrin shown in **Figure 2** (26). Notably, priming signal such PAMPs and DAMPs are essential initiation factors of NLRP3 activation. When the priming signals bind to membrane-bound (TLRs, TNF-R, and IL-1R) receptors, they modulate the intracellular signal transduction pathway that ultimately activates MAPK and the transcription factors such as NF-κB and AP-1, which are essential for the translocation of inflammasome components from the nucleus into the cytosol in both innate immune and non-immune cells. A recent finding suggests that PI3K/AKT-dependent NLRP3 inflammasome complex formation was attenuated by the PI3K inhibitor more effectively than the AKT inhibitor during the inflammatory process (27).

**Figure 3:**
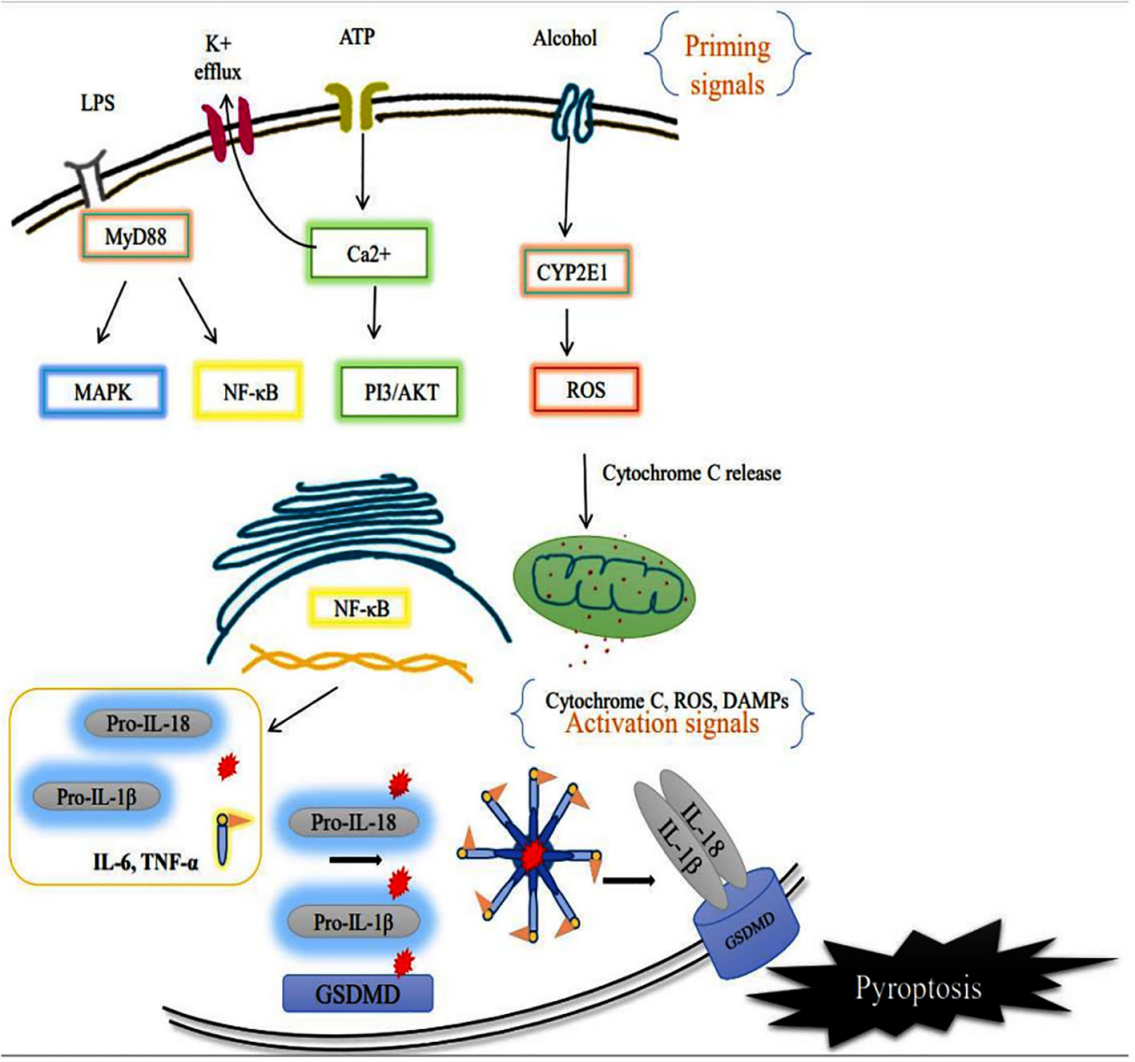
Activation of membrane receptor (priming signal) leads to acceleration of the intracellular NF-κB signaling pathway that translocates the inflammasome components such as NLRP3 (sensor), and caspase-1 to the cytosol. CYP2E1 mediated ROS production alters the intercellular organelle integrity as well as mitochondrial dysfunction via cytochrome C release. The endogenous factors/secondary signals such as cytochrome C, ATP, ROS act as mediators of NLRP3 inflammasome complex formation, and facilitate autoproteolytic cleavage of caspase-1 which, in turn, cleaves the pro-interleukins IL-1β, IL-18 and Gasdermin D (GSDMD). Ultimately the cell undergoes pyroptotic cell death, further worsening the tissue microenvironment.

The sensor component of the NLRP3 inflammasome could recognize various exogenous and endogenous stimuli such as Ca^+^ influx, K^+^ efflux, mitochondrial damage, ROS, extracellular ATP, and gut microbial components (28). Priming signal is a crucial step that translocate the NLRP3 from the nucleus to the cytosol. While the sensor further gets activated through various endogenous factors such as cytochrome C and ROS, termed the activation signals. This secondary signal or activation signal associated with the NLRP3 (sensor) modulates the confirmation that recruits the adaptor ASC via homotypic interaction of pyrin-pyrin and CARD-CARD domains. This aids in the assembly of these three essential components and their aggregation that results in NLRP3 inflammasome complex formation (29). Upon activation, the autoproteolytic cleavage of p20 and p10 subunits from the CARD domain of pro-caspase-1 results in the formation of active caspase-1. A functional form of IL-1 beta-converting enzyme (ICE), or caspase-1, cleaves the immature pro-interleukin-1β and pro-interleukin 18 into biologically active IL-1β and IL-18. It also cleaves the cytoplasmic protein gasdermin-D from its autoinhibitory C-terminal domain. This allows the gasdermin-D N-terminal domain to bind with membrane phosphatidylinositol phosphates, phosphatidylserine, and cardiolipin, and oligomerize to form pores, that alter the osmotic potential and release active pro-inflammatory cytokines to the extracellular matrix (a process termed pyroptosis) (30). A recent study reports that a member of the interferon regulatory factor family (IRF2; transcription factor) is necessary for the post-transcriptional modification of the gasdermin-D gene (31). Most strikingly, IRF-2 deficiency significantly inhibits the caspase-1 dependent IL-1β secretion and pyroptosis in macrophages, epithelial cells, and other tissue-resident cells (31). Further, pyroptosis-produced cytokines and chemokines bind to their cognate receptors on the immune cells like leukocytes and lymphocytes to activate the signal transduction pathways such as NF-κB and MAPK that enhance pro-inflammatory cytokines such as TNF-α, IL-6, and IL-17 as well as chemokines to induce the inflammatory response (32–34). Aberrant activation of the NLRP3 inflammasome-mediated release of pro-inflammatory cytokines from hepatocytes recruits peripheral mononuclear cells to the microenvironment and promotes inflammation and tissue damage (35). As suggested, inflammasomes exist in parenchymal and non-parenchymal cells of liver and are emerging as pathogenic mediators in ALD as well as non-alcoholic fatty liver disease. The formation of the NLRP3 inflammasome complex plays a significant role in the pathogenesis and progression of ALD through the excessive production of pro-inflammatory cytokines as shown in **Figure 3**. Expression of IL-1β and IL-18 cytokines is highly elevated in the hepatocytes of patients with ALD (36).

Although other inflammasomes such as NLRP1, NLRP6 and NLRP12 regulates the progression of liver diseases, NLRP3 inflammasome is considered as the potent modulator of the pathophysiological function in ALD (37). It is yet unknown how NLRP1, 2, 6 and 12 is involved in chronic alcoholic liver disease or other liver conditions, there are few limited studies have been done but none on ALD. Definitive understanding of the mechanism of inflammasomes in the liver is necessary for the development of inflammasome-based therapies for ALD.

## 3 Ethanol promotes NLRP3 activation in ALD

NLRP3 inflammasome activation by ethanol, plays an important role in the pathogenesis of ALD, and the profound studies on this mechanism has contributed to the progress of new therapeutic strategies for ALD. Overall the NLRP3 inflammasome aggravates the inflammatory signaling cascade with its persistent pro-inflammatory mediators. There are no studies that specifically highlight the role of ethanol metabolites in the priming or activation of the NLRP3 pathway. Also, the existing finding results that it could enter either directly through the gut-liver axis or indirectly by actively crossing the membrane (38).Chronic alcohol exposure activates CYP2E1 in hepatocytes, resulting in the dysfunction of antioxidant system along with excessive production of ROS and inducible nitric oxide synthase (iNOS) (39, 40), which cause ER stress and activate inflammatory response via TLR4/MyD88/NF-κB signaling axis to significantly promote NLRP3 inflammasome activation (41, 42). At the same time, the release of a pro-inflammatory signal ultimately provokes the infiltration of non-resident immune cells and activation of resident immune cells. An earlier study stressed that the translocation of gut-microbial content (LPS) and cellular debris (ATP, uric acid, and HMGB1) promotes the inflammasome activation, which mediates inflammatory markers, that are measured as the pathogenic mediator of ALD (43). During ethanol treatment, hepatocyte-derived ATP binding with P2X7 (purinoceptor 7) initiates inflammasome formation and facilitates pyroptotic cell death (44).

Reactive oxygen species (ROS) generation is one of the major events during alcohol induced disease progression. Alcohol metabolism via alcohol dehydrogenase and microsomal cytochrome P450 2E1 results in increased ROS generation in the liver (45). The imbalance between ROS generation and detoxification via several enzymes such as superoxide dismutase (SOD), *γ*-glutamyltransferase (GGT), glutathione (GSH), glutathione reductase (GSSG-Rd), glutathione peroxidase (GSH-Px), glutathione S-transferase (GST), and catalase (CAT) causes oxidative stress (46). Excessive production of ROS activates NLRP3 inflammasomes, which, in turn produces more ROS via oxidized mitochondrial DNA (47). Mitochondrial ROS acts as a secondary stimulus for the NLRP3 inflammasome activation that leads to the activation of caspase-1 and production of pro-inflammatory cytokines IL-1β and IL-18 (48). Recent research has shown that thioredoxin-interacting protein (TXNIP), an endogenous regulator of redox/glucose-induced stress and inflammation, plays a role in the activation of NLRP3 inflammasome (49). Expression of inflammasome components such as NLRP3, ASC, and caspase-1 was higher in ethanol-fed mice than in WT mice (50).

In contrast, lack of the NLRP3 inflammasome component reverses the increased secretion of pro-inflammatory cytokine mediated steatosis and liver damage in alcoholic mice (51). The serum of ALD patients show a significant increase in the expression of LPS and ATPs, with uric acid levels comparable to healthy controls (52). Surprisingly, one-time alcohol intake by healthy subjects also showed identical results to those of ALD patients (14). In addition, an *in vivo* study demonstrates that the expression of the NLRP3 protein was upregulated in the liver of alcohol-fed mice. In contrast, increased activation of NF-κB associated with the lack of caspase-1 and/or other components of the inflammasome prominently reduced pro-inflammatory cytokines such as IL-1β and IL-18 in hepatocytes (13). IL-1β binds to IL-1 receptor (IL-1R), which induces Kupffer cell activation and thus leads to hepatitis, cellular injury, and fibrosis. Treatment with anakinra (IL-1R1 antagonist) attenuates the intracellular pro-inflammatory signaling cascade that reciprocates alcohol-induced liver damage (53). Further, the spleen tyrosine kinase (SYK) regulates inflammasome activation through phosphorylation of ASC (54). Levels of activated SYK were elevated in the livers of alcohol-fed mice (54). The inhibition of SYK in alcohol-fed mice remarkably decreased the serum levels of IL-1β and caspase-1 activation in the liver. Inhibiting the inflammasome component with commercially available antagonists and target-specific therapy will probably negate the progression of liver disease. Currently, the adverse effects of the NLRP3 inflammasome activations could be mitigated by treatment with anakinra (an IL-1R1 antagonist), allopurinol (a uric acid synthesis inhibitor), or probenecid (a uric acid reducer and ATP signaling blocker) in an experimental study involving the ALD condition (55). In several clinical studies, MCC950, a synthetic antagonist of NLRP3, has shown the efficacy to halt the process of NLRP3 inflammasome complex formation, and decreased inflammation by preventing pyroptotic cell death (56). Few clinically approved NLRP3 inhibitors are listed in **Table 1**.

**Table 1:** Potent inhibitors available for the NLRP3 inflammasome and its components.

Inhibitor	Inhibition Mechanism	Specificity	Clinical Status	References
MCC950	Interacts with Walker B motif, prevents ASC oligomerization	NLRP-3	Phase 2	(57)
CY-09	Interacts with Walker A motif, prevents ASC oligomerization	NLRP-3	–	(58)
Glibenclamide	Blocks ATP-sensitive K^+^ channels	NLRP-3	Phase- 4	(59)
Parthenilide	Caspase 1 and ATPase inhibitor	NLRP-3, AIM2, NLRP-1	–	(60)
Bay11-7082	ATPase inhibitor	NLRP3	–	(61)
Emricasan	Caspase inhibitor	NLRP3	Phase 3	(62)
β- Hydroxybutyrate	K^+^ Efflux inhibition, prevents ASC oligomerization	NLRP3	–	(63)
Andropgrapholide	NF- κB inhibitor	AIM2	–	(64)
Necrosulfonamide	GSDMD inhibitor	NLRP3	Phase 2	(65)

"-" symbol representing there is no clinical status available.

### 3.1 Ethanol related epigenetic modifications on inflammasomes in ALD: an emerging therapeutic target

Evidence shows that epigenetics plays an imperative role in the pathogenesis of ALD. Here, we review recent findings on the epigenetics of ALD and its potential therapeutic strategies. Epigenetics is a feature of chronic inflammation in many chronic illnesses (66). Epigenetic regulation is traditionally defined as the process causing possible heritable changes in gene expression, however, without altering the DNA sequences. These modifications can be accomplished by at least three mechanisms: CpG DNA methylation, histone post-translational modifications (PTMs), and noncoding RNA production (67).

**Figure 4:**
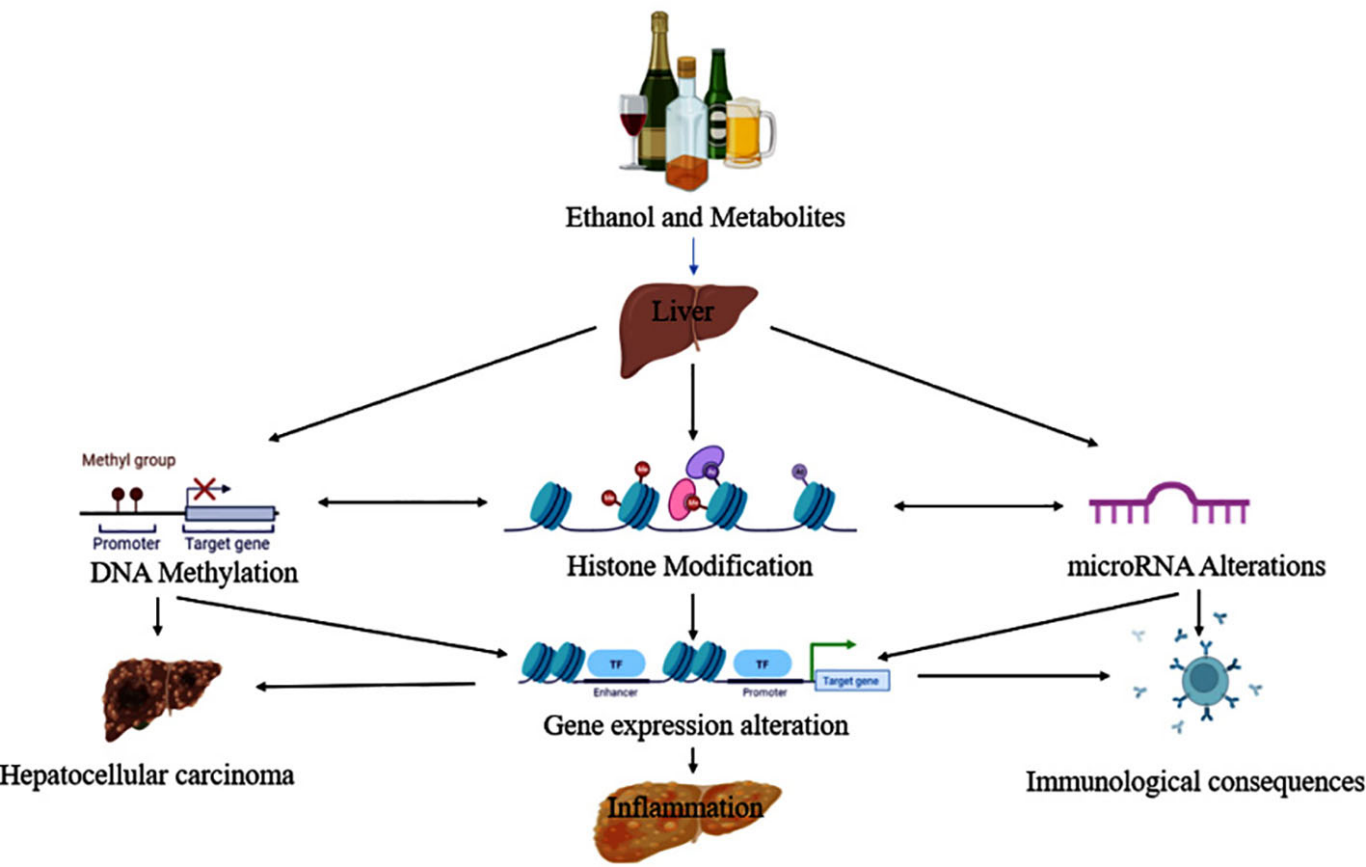
Ethanol and its metabolites lead to the progression of liver disease via epigenetic modifications.

Park and colleagues were the first to report the pathological evidence in ethanol-induced epigenetic changes in histone H3 proteins. Additional reports later showed that ethanol altered histone H3 methylation at two lysine residues (*lys-4 and lys-9*), and phosphorylation at two serine residues (*ser-10 and ser-28*) (68). The histone proteins combine to form bigger complexes known as nucleosomes, which wrap the DNA in the cell nucleus. Modifications to histone H3 at various places (e.g., *lys-4, lys-9, ser-10, ser-28*, etc.) may occur on nucleosomes in the same or separate domains of the chromatin. These site-specific modifications, in turn, connect themselves with changes in gene expression. Epigenetic changes and their association with diverse pathological consequences from ethanol exposure are depicted in **Figure 4** (69).

Significant efforts have been undertaken in recent years to uncover the epigenetic mechanisms that govern the expression of NLRP3 inflammasome components (direct mechanisms) or the essential regulators involved in these assembly and activation (indirect mechanisms) (19). DNA methylation occurs when a methyl group is added to the fifth carbon position of the pyrimidine ring of cytosine residues at the level of CpG dinucleotides and leads to transcriptional suppression (70). The existence of elevated DNA methylation levels in the promoters of ASC, caspase 1, and IL-1β genes in human monocytes indicate the significant role of DNA methylation in the epigenetic regulation of NLRP3 inflammasome components, and alternatively the hypomethylation in these regions enable them to be expressed (71). Numerous studies have shown the importance of DNA methylation is in controlling the NLRP3 sensor. According to Wei et al. Mycobacterium tuberculosis-infected THP-1 cells that had differentiated into macrophages showed considerably lower levels of DNA methylation in the NLRP3 gene promoter than did uninfected cells. As a result, NLRP3 expression is increased, inflammasomes are activated along with pro-inflammatory cytokines IL-1β and IL-18 are released in response to infection (72). Similarly, modifications in DNA methylation levels also influence the ASC adaptor molecule. Although the results are inconsistent and depend on the kind of tumor, the ASC gene is known to be hypermethylated in a variety of tumor types, where it is related with prognosis (73, 74). In various tumor, including non-small cell lung cancer, gastric cancer, renal cell carcinoma, and lung adenocarcinoma, hypermethylation of ASC is linked to worse survival rates and advanced disease stages. Patients with glioblastoma, gastric cancer, and oral squamous cell carcinoma have shorter overall survival times, larger tumor, and deeper tumor when ASC is overexpressed and its methylation has been lost (75, 76). Wu et al. research (77) demonstrated a correlation between high levels of protein expression in oral cavity squamous cell carcinoma tissues and low DNA methylation levels in the ASC gene. Additionally, elevated amounts of IL-1β, CASP-1, and NLRP3 mRNA were found, suggesting that the NLRP3 inflammasome plays a part in the pathogenesis. ASC overexpression promotes metastasis by enhancing cell invasion and migration. Notably, H3K27me3 and H3K4me3 deposition at the promoter region of the genes involved in the identity and differentiation of neural precursor cells decreased as a result of alcohol exposure. Whereas altered methylation was reported in ethanol treated rat hepatocytes, with increased H3K9 and decreased H3K4 dimethylation (77). Although further verification is essential, this epigenetic modification may partly contribute the process of ALD.

Accumulating studies have revealed that acetylation dynamics are the primary regulatory mechanism causing NLRP3 inflammasome activation (19). Acetylation of specific histone residues by histone acetyltransferases (HATs) not only allows the formation of an open transcribable chromatin structure accessible to transcriptional factors, but also facilitates the binding of epigenetic readers capable of recruiting all of the transcriptional machinery required to initiate RNA polymerase II activation (78). Changes in the acetylation dynamics of genes implicated in the NF-κB pathway affect transcription of NLRP3, ASC, Caspase-1, and IL-1β (79)

Additionally, the PYD domain of the NLRP3 protein in macrophages has lysine residues that have been acetylated (80). Inflammasome activation and assembly with ASC are mediated by NLRP3 acetylation in response to LPS as well as ATP. Conversely, NLRP3 inflammasome activity is suppressed by the loss of NLRP3 acetylation caused by the SIRT2 NAD^+^-dependent deacetylase. Aged SIRT2-deficient mice or mice given a high-fat diet displayed elevated plasma levels of IL-18, insulin, and glucose, which is associated with elevated NLRP3 inflammasome activation. As a result, NLRP3 deacetylation caused by SIRT2 overexpression decreases inflammasome activation in macrophages and guards against chronic inflammation and insulin resistance linked to aging. Similarly, SIRT3 loss in diabetic cardiopathy-affected rats enhances NLRP3 expression, which encourages caspase-1 activation and the maturation and cleavage of the pro-form of IL-1β (81). According to previous studies, acute ethanol exposure impacts on the epigenome by activating histone acetyltransferases (HATs) and inhibiting histone deacetylases (HDACs) in the rat amygdala brain regions which results in chromatin remodeling and alterations in gene expression (82–85).Previous studies have also demonstrated that various alcoholic beverages and their metabolites can also alter the acetylation of histones in hepatocytes which leads to ALD (86).

MicroRNAs (miRNAs) are epigenetic modulators that decrease the expression of target genes post-transcriptionally. Several miRNAs have been shown to bind to the 3′-untranslated regions of NLRP3 gene thus encouraging its degradation. During the development of liver diseases, downregulation of certain miRNAs (miR-223, miR-7, miR-30e, miR-22, and miR-495) or hypermethylation of miR-145 leads to increased expression of all inflammasome components and subsequent NLRP3 inflammasome activation (76–80, 87–90). In addition to the previously described epigenetic regulation mechanisms that directly target the expression of various NLRP3 inflammasome components, epigenetic changes in transcription factors such as nuclear facto r-erythroid 2 -related factor (Nrf2), ROS production modulators thioredoxin-interacting protein (TXNIP) and mitochondrial uncoupling protein 2 (UCP2), genes encoding microtubule stabilization proteins - microtubule affinity regulating kinase 4 (MARK4), and autophagy gene (ATG5) could facilitate changes in the expression of NLRP3 inflammasome components which are involved in inflammasome activation (81–83, 91). Focusing on the upregulation of these epigenetic factors through various drugs would be a promising therapeutic approach to halt the persistent activation of NLRP3 inflammasome. For instance, miR-148a inhibits TXNIP which, in turn, fortify against the assembly of inflammasome and pyroptosis (92–94). *Lv Qi et al.* reported that the treatment with lonicerin enhances the lysosomal degradation of NLRP3 inflammasome by upregulating the ATG5 gene in C57BL/6 mice (95, 96).

Epigenetic modulation on the upstream of inflammasome activation boosts the inflammation process. ROS-mediated oxidative stress, an inflammasome activator, affects chromatin remodeling by altering histone acetylation and deacetylation events (37, 86). Alcohol-induced acetylation of histone H3 in rat hepatocytes is mediated via ROS. Inhibiting NADPH-oxidase-mediated ROS reduces H3AcK9, while ROS inducers directly increase alcohol-induced acetylation of H3K9 (97). With the stimulation of intrinsic HAT activity, oxidative stress activates NF-κB, resulting in the production of pro-inflammatory mediators. Furthermore, oxidative stress decreases HDAC activity, which leads to a persistent inflammatory response followed by promoting a complex formation between the coactivator CBP/p300 and the NF-κB p65 subunit, implying a function for oxidative stress in chromatin remodeling (98). Thus, targeting the inflammasome activators in the epigenetic level can reverse the process of inflammasome-mediated ALD.

Thus, the hypothesis is that targeting epigenetic modifications related to inflammasome and its components could possibly reduce the progression of ALD. In recent times, several epidrugs such as DNMT inhibitors, HAT inhibitors, and HDAC inhibitors are used in routine clinical practice to subside the progression of various diseases (summarized in **Tables 2**–**4**). Use of these epidrugs could reduce the features of epigenetic modifications in NLRP3 inflammasome, thereby ameliorating ALD. Results from studies on epidrugs in various diseases suggest that targeting NLRP3 epigenetic modification may serve as a novel strategy to prevent ALD. Given that the overactive NLRP3 inflammasome has a pathogenic role in ALD, it is a prospective therapeutic target for ALD. Indeed, a large number of inhibitors (**Table 1**) that target the pathways upstream and downstream of the NLRP3 inflammasome have recently been discovered.

**Table 2:** Methylation inhibitory drugs.

Inhibitor	Inhibition mechanism	References
5-Azacytidine	Degradation of DNMT1 enzyme	(99)
Zebularine	Degradation of DNMT1 enzyme	(100)
MG-98	Prevent the transcription of *DNMT1* gene	(101)
RG108	Binds to the active site of DNMT1	(102)

**Table 3:** HAT inhibitory drugs.

Inhibitor	Inhibition mechanism	References
Curcumin	Inhibition of histone H3 and histone H4 acetylation	(103)
Garcinol	Inhibition of p300 acetylation	(104)
Isothiazolones	Inhibition of p300 and PCAF	(105)
Lys-CoA	Inhibition of p300	(106)

**Table 4:** HDAC inhibitory drugs.

Inhibitor	Inhibition mechanism	References
Vorinostat	Binds to catalytic site of HDAC	(107)
CY-1215	Targets HDAC6	(108)
Romidepsin	Inhibits enzymatic activity of HDAC 1 and 2 by interaction with zinc ion of catalytic site	(109)

#### 3.1.1 The NLRP3 inflammasome activation pathway’s downstream inhibitors

##### 3.1.1.1 IL-1 targeting drugs

The principal effectors of an active NLRP3 inflammasome are IL-1β and IL-18, which cause pathogenic alterations in a variety of autoimmune disorders. When IL-1β binds to IL-1R1 on different immune cells, IL-1R accessory protein (IL-1RAcP) causes receptor heterodimerization. This initiates the NF-κB and MAPK cascades, intracellular MyD88, and eventually the NLRP3 inflammasome signaling (110). Currently, three anti-IL-1 drugs have received clinical approval for use: Anakinra, a recombinant version of the naturally occurring IL-1Rα, canakinumab, a monoclonal antibody that neutralizes human IL1β, and rilonacept, a soluble decoy receptor that blocks binding of IL-1β or IL-1α to IL-1R1 (111).

##### 3.1.1.2 Inhibitors for NLRP3

The importance of NLRP3 in autoimmune disorders and its strong propensity for inflammation make it a desirable therapeutic target. Several small chemical inhibitors targeting oligomerized NLRP3 protein have been created in recent years, including MCC950, JC-171, OLT1177, CY-09, MNS, and Bay 11-7082, since NLRP3 oligomerization is a crucial step for anchoring the ASC protein and subsequent activation of the inflammasome (110, 112).

Coll and colleagues initially identified MCC950 as a highly selective small molecule inhibitor of the NLRP3 inflammasome, which is now a common research tool (57). By directly attaching to the Walker B motif in the NACHT domain, MCC950 targets the ATPase activity of the NLRP3 inflammasome. This prevents ATP hydrolysis, NLRP3 protein oligomerization, and inflammasome formation (113). Another recently developed drug JC-171 is a glyburide analogue that targets the NLRP3-ASC interface to reduce NLRP3 inflammasome activity and IL-1β secretion (114). An efficient and targeted NLRP3 inflammasome inhibitor, OLT1177 said to have little effect on NLRC4 or AIM2 inflammasomes while drastically inhibiting the activity of NLRP3 inflammasome. OLT1177 can prevent the secretion of mature IL-1β and IL-18 without changing the production of the precursor protein, regardless of the canonical or non-canonical activation route. Considering the mode of action, OLT1177 can either bind to the ATPase of the NLRP3 NACTH domain or stop NLRP3 from interacting with ASC (115). Other than these analogues there are some natural compounds which can inhibit NLRP3 for example Oridonin, a naturally occurring substance obtained from the therapeutic plant *Rabdosia rubescens*, binds to cysteine 279 of NLRP3 in the NACHT domain by covalent bonding and prevents NLRP3 and NEK7 from interacting, preventing the activation of inflammasomes (116). Despite the positive outcomes of preclinical investigations, biosafety concerns have prevented most NLRP3 inhibitors from moving on to clinical trials. Nevertheless, more study and development will be required since the NLRP3 inflammasome represents a viable therapeutic target for ALD.

##### 3.1.1.3 Inhibitors for *Caspase-1*

A critical step in the secretion of IL-1β and IL-18 is caspase-1 cleavage, inhibiting caspase-1 can also prevent the activation of the NLRP3 inflammasome followed by subsequent autoimmune reactions. Peptidomimetic inhibitors VX-740 and its analogue VX-765 by covalently altering the cysteine residues at the active site of caspase-1, prevents the enzyme from performing its normal function (56) and consequently ameliorate inflammation and improve the immune microenvironment. Nevertheless, clinical trials for these inhibitors were terminated due to its hepatotoxicity (117).

##### 3.1.1.4 Inhibitors for *GSDMD*

By creating pores in the cell membrane, cleaved-GSDMD has a significant impact on the release of proinflammatory cytokines and other variables. GSDMD is a particularly desirable therapeutic target since it is involved in the last stage in the activation and functioning of all inflammasomes. Inhibiting GSDMD can prevent the release of cytokines linked to inflammasomes and reduce the signs and symptoms of inflammation (110). FDA approved alcohol deterrent, disulfiram, is used to treat withdrawal symptoms in alcohol addiction, suppresses GSDMD-mediated pyroptosis *in vitro* by inhibiting pore formation and liposome leakage, which in turn reduces IL-1β and IL-18 production by activated NLRP3 inflammasome (118).

On the other hand, as discussed previously epigenetic modifications too may lead to ALD thus, the hypothesis is that targeting epigenetic modifications related to inflammasome and its components could possibly reduce the progression of ALD. In recent times, several epidrugs such as DNMT inhibitors, HAT inhibitors, and HDAC inhibitors are used in routine clinical practice to subside the progression of various diseases (summarized in **Tables 2**–**4**). Use of these epidrugs could reduce the features of epigenetic modifications in NLRP3 inflammasome, thereby ameliorating ALD. Results from studies on epidrugs in various diseases suggest that targeting NLRP3 epigenetic modification may serve as a novel strategy to prevent ALD.

## 5 Concluding remark

The inflammatory response induced by NLRP3 inflammasome activation is critical in the development of ALD. The major inflammatory signals in liver pathology are NLRP3 inflammasome-induced cytokine release and pyroptosis. In this review, we summarized the involvement of the NLRP3 inflammasome and its components in the pathogenesis of ALD. Chronic unresolved inflammation is a key driver of disease progression in ALD and is one of the most promising treatment targets. Over the last decade, significant progress has been made in understanding the role of NLRP3 inflammasome formation and activation in the onset and progression of liver injury, as well as the cell-specific contribution to both upstream and downstream signaling pathways involved. Targeting inflammasome is one of the crucial steps in case of disease progression. As epigenetics and its relevance are gaining increased importance in disease pathology, for diagnosis, and control, much efforts are needed to understand how epigenetics mechanisms may be targeted to attenuate ALD. Available evidence suggests that epidrugs may be helpful in altering inflammasome formation and activation which, in turn, may attenuate the progression of ALD. However, further studies are needed to establish the link between inflammasome activation and epigenetic regulation in ALD to harness this pathway to control ALD at the beginning stage itself.

## Author contributions

RT and VM outlined and the structured review; SB and TD collected related studies and wrote the first draft of the manuscript; TD and SB created the figures and tables; RT, VM, and VS proofed the text and figures. VM, VS, and RT revised the manuscript. All authors contributed to the article and approved the submitted version.
